# Regional Brain Localization of Botulinum Toxin Type A-Truncated Synaptosomal-Associated Protein 25 After Injection into the Rat Hind Paw

**DOI:** 10.3390/toxins18060261

**Published:** 2026-06-09

**Authors:** Dalia Nemanić, Mihael Grdunac, Petra Šoštarić Mužić, Patrik Meglić, Ivica Matak, Lidija Bach-Rojecky

**Affiliations:** 1 Department of Pharmacology, University of Zagreb, Faculty of Pharmacy and Biochemistry, A. Kovačića 1, 10000 Zagreb, Croatia; dalia.nemanic@pharma.unizg.hr (D.N.); mihael.grdunac@pharma.unizg.hr (M.G.); lidija.bach@pharma.unizg.hr (L.B.-R.); 2 Department of Neuroscience, Karolinska Institutet, Solnavagen 9, Kvarter B4, 17165 Solna, Sweden; petra.sostaric.muzic@ki.se; 3 Laboratory of Molecular Neuropharmacology, Department of Pharmacology, Croatian Institute of Brain Research, University of Zagreb, School of Medicine, Šalata 11, 10000 Zagreb, Croatia; patrik.meglic@mef.hr (P.M.); ivica.matak@mef.hr (I.M.)

**Keywords:** botulinum toxin type A, trans-synaptic transport, brain distribution, BoNT-A-neutralizing antitoxin, immunohistochemistry, cleaved synaptosomal-associated protein of 25 KDa

## Abstract

We previously demonstrated that botulinum neurotoxin A (BoNT-A) exerts bilateral antinociceptive effects, involving trans-synaptic transport at the level of the lumbar spinal cord. However, the potential distribution of the toxin to supraspinal sites has not yet been investigated. In the present study, we examined the distribution of cleaved SNAP-25 (cl-SNAP-25), a marker of BoNT-A activity, in the rat brain following peripheral unilateral BoNT-A administration. Brain tissues from rats treated with BoNT-A (7 U/kg, into the hind paw) were analyzed using immunofluorescent tyramide signal amplification to detect cl-SNAP-25. To assess the contribution of trans-synaptic transport, a BoNT-A-neutralizing antitoxin (2 IU) was administered intrathecally 24 h after BoNT-A injection. Signal intensity was evaluated using a semi-quantitative immunohistochemical scoring method based on cl-SNAP-25-positive nerve fibers. Bilateral cl-SNAP-25 immunoreactivity was observed in multiple supraspinal regions, most prominently within the trigeminal complex and the facial and gracile nuclei. Signal intensity was significantly reduced by intrathecal antitoxin, indicating that trans-synaptic transport contributes to central BoNT-A distribution. Peripherally administered BoNT-A reaches distant supraspinal regions, possibly via neuronal retrograde and trans-synaptic transport. Further studies are warranted to clarify exact pathways and alternative distribution routes, determine the functional relevance of central BoNT-A presence, and assess its clinical implications.

## 1. Introduction

Botulinum neurotoxin type A (BoNT-A), produced by anaerobic bacterium *Clostridium botulinum*, is one of the deadliest toxins known in nature (LD_50_ = 0.5–1 ng/kg), and the most prevalent BoNT serotype involved in human botulism [1]. Its effects are primarily mediated by impaired synaptic acetylcholine release from peripheral motor and autonomic nerve terminals that induce flaccid paralysis of skeletal muscles and dysautonomia. Inside synapses and along neuronal processes, BoNT-A cleaves synaptosomal-associated protein of 25 kDa (SNAP-25), its only known biological target, thus interfering with neurotransmitter exocytosis [2]. It is commonly used as a pharmaceutical preparation derived from a BoNT-A subtype A1-producing Hall strain containing 900 kDa complex, consisting of 150 kDa neurotoxin component and 750 kDa non-toxic auxiliary proteins (INN: *Clostridium botulinum* type A neurotoxin complex) [3]. As such, it is one of the most widely used therapeutic proteins for spasticity and dystonia, different autonomic disorders (hyperhidrosis, neurogenic bladder), and for pain treatment (migraine, off-label use in different neuropathic pain conditions). The mainstay of its treatment strategy remains targeting the exact hyperactive nerve endings in the periphery that provokes the underlying medical condition, supported by precision-guided injections (e.g., by combining ultrasound and EMG to target muscles) [4].

Despite its long-standing clinical use (for more than 35 years), relatively little is known about all the potential sites of BoNT-A actions. Therapeutic use, as well as peripheral side effects of therapeutically recommended toxin doses, ranging from around 100 pg (corresponding to 2 U defined in terms of mouse LD_50_) for spasmodic dysphonia to around 20 ng (corresponding to 400 U, which represents approx. 1/5 of its lethal dose), involves toxin actions near the injections sites, with local distribution by passive diffusion within the injected tissue. However, even moderate toxin doses in some patients can lead to distant side effects related to systemic spread of the toxin [5]. Subclinical changes in the distant muscle physiology, such as the increased jitter [6], suggest systemic toxin spread even after low-toxin therapeutic doses.

In addition, an increasing amount of preclinical and clinical data suggests that previously unrecognized, albeit limited, toxin trafficking to the central nervous system (CNS) can occur, with important implications for its therapeutic efficacy in pain and hyperkinetic movement disorders. Numerous preclinical studies, from behavioral experiments [7,8,9] to immunohistochemical analyses of cl-SNAP-25 protein [10,11,12,13], have demonstrated that the antinociceptive effect of BoNT-A involves its retrograde axonal transport from the peripheral administration site to the CNS. After the toxin’s unilateral application, the cl-SNAP-25 expression was detected at the level of the first synapse (e.g., spinal cord segments), not only on the injected side, but on the contralateral side as well. These findings supported behavioral experiments showing that BoNT-A reduces pain even on the side opposite to its unilateral application. It was hypothesized that bilateral effects could result from the toxin’s trans-synaptic transport between the cells at the level of the first synapse [9,14,15]. Early experiments in the late 1970s using radiolabeled BoNT-A, supported by neurophysiological studies, revealed that intramuscularly injected BoNT modifies neuronal activity in the spinal cord of rats and cats. These findings implied that retrogradely transported BoNT-A can affect higher-order neurons via trans-synaptic passage. In the 1990s, a series of studies provided further compelling functional evidence supporting the trans-synaptic passage of BoNT/A in experiments in cats (reviewed in [16]). In experiments by Antonucci et al. [17], cleaved SNAP-25 was detected at retinal synapses following BoNT-A injection into the rat superior colliculus, thus suggesting trans-synaptic transport of catalytically active BoNT-A within the rat visual system. Furthermore, several in vitro experiments on cultures of motor [18] and hippocampal neurons [19,20] demonstrated the toxin’s ability to leave the primary neurons and enter the upstream neurons. The experiments that followed further supported BoNT-A trans-synaptic transport within the animal motor [21,22] and sensory system [14,15] at the level of the first synapse in the spinal cord.

Clinically observed distant effects, such as the remote F-wave or recurrent inhibition changes in non-injected muscles [23,24], cannot be explained by peripheral or systemic distribution after toxin intramuscular application, but rather by its axonal and possibly trans-synaptic spread within spinal cord central circuits and projections.

In line with that, studies on potential remote central sites of toxin action are of utmost importance, as these may result from either (a) direct axonal transport or trans-synaptic spread through projection neurons from the spinal cord or brainstem linked to the injected peripheral site, or (b) diffusion combined with systemic dissemination to various body regions, followed by axonal transport via sensory or motor neurons to primary sensory or motor areas.

Recently, we showed that a single unilateral peripheral injection of BoNT-A (7 U/kg) into the rat hind paw causes bilateral antinociceptive effects on carrageenan-induced bilateral mechanical hyperalgesia that included trans-synaptic toxin transport to the contralateral side of the spinal cord [15]. Here we extend these experiments by analyzing the brain tissue obtained from the mentioned study to screen for cl-SNAP-25 occurrence in different brain regions. Therefore, the objectives of the present study are to investigate the potential distribution of BoNT-A in supraspinal regions following peripheral administration and to explore the contribution of trans-synaptic transport to its distribution within the CNS.

## 2. Results and Discussion

By employing the immunofluorescent tyramide signal amplification method, here we examined the presence of the cl-SNAP-25 in the supraspinal regions after unilateral peripheral BoNT-A application (7 U/kg, i.pl. into the hind-paw pad). Intrathecally applied BoNT-A-neutralizing antitoxin (2 IU, i.t., 24 h following BoNT-A) was employed to elucidate if the cl-SNAP-25 occurrence depends on its trans-synaptic transport between neurons. Previously, we examined the distribution of cleaved SNAP-25 in second-order sensory brain regions following BoNT-A peripheral application by employing Alexa-labeled secondary antibodies and were unable to confirm any toxin distribution beyond Sp5C [25]. However, the detection of the signal was hampered by low signal intensity that was best visible only in high magnification, making the visual signal detection, particularly in low-intensity fibers, difficult. In contrast to Alexa-labeled secondary antibodies, tyramide signal amplification has enabled superior signal intensity and signal-to-noise ratio [13,22]. The observed signal is not a result of background staining but rather reflects specific labeling of neuronal structures (Supplementary Figure S1. Controls).

### 2.1. Cleaved SNAP-25 Occurs Bilaterally in Trigeminal Subnuclei and Is Attenuated by BoNT-A-Neutralizing Antitoxin

**Figure 1:**
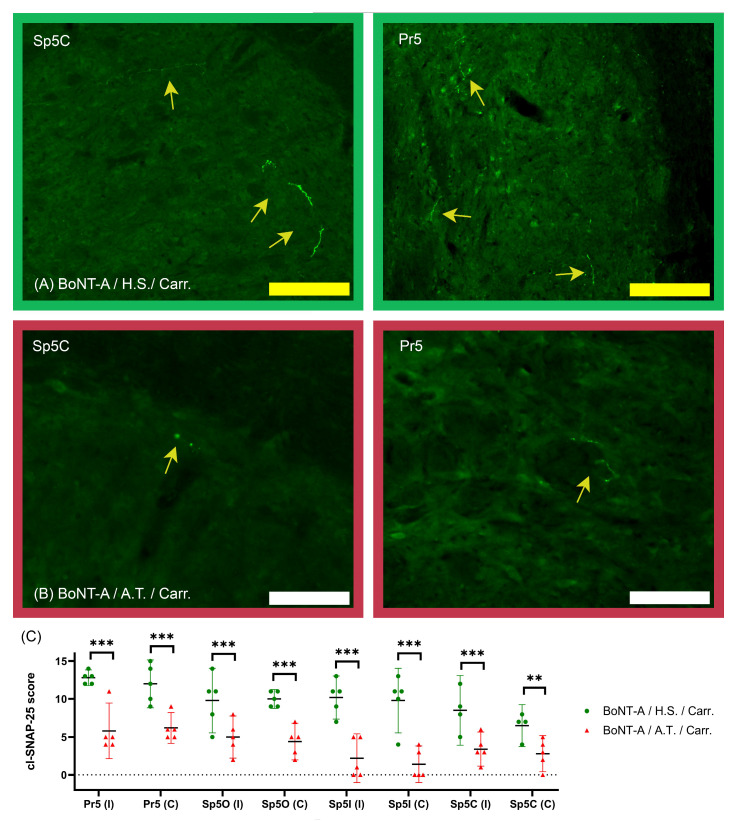
Peripheral BoNT-A induces cl-SNAP-25 immunoreactivity within the trigeminal complex that is reduced by BoNT-A-neutralizing antitoxin treatment. (**A**) Representative images of the ipsilateral BoNT-A proteolytic activity (cl-SNAP-25; green immunostaining indicated by arrows) in the caudal subnucleus of trigeminal complex (Sp5C) and the principal sensory nucleus (Pr5) after unilateral peripheral BoNT-A administration. (**B**) Antitoxin treatment reduced cl-SNAP-25 immunoreactivity in both regions. Images outlined in green correspond to animals treated with BoNT-A alone, whereas those in red correspond to animals receiving BoNT-A with antitoxin. White scale bar = 100 µm; yellow bar = 200 µm. (**C**) Semi-quantitative analysis of cl-SNAP-25 immunoreactivity in the Pr5, Sp5O, Sp5I, and Sp5C. For each animal (N = 5), values were obtained by summing up mean scores from three independent observers. Results are expressed as mean with 95% CI. Data were analyzed using linear mixed-effects models. *p*-values were adjusted for multiple comparisons using the two-stage linear step-up procedure of Benjamini, Krieger, and Yekutieli to control the false discovery rate (FDR < 0.05). Adjusted *p* < 0.05 was considered statistically significant. ** = *p* < 0.01; *** = *p* < 0.001. Figures were processed for brightness and contrast adjustments using Adobe Photoshop 2021 (version 22.0.0; Adobe Systems Incorporated, San Jose, CA, USA). Abbreviations: Pr5 = principal sensory nucleus; Sp5O = oral trigeminal subnucleus; Sp5I = interpolar trigeminal subnucleus; Sp5C = caudal trigeminal subnucleus; I = ipsilateral; C = contralateral; H.S. = horse serum; A.T. = antitoxin to BoNT-A; Carr. = carrageenan.

Cleaved SNAP-25 was detected throughout the trigeminal complex, including the principal sensory nucleus (Pr5), oral subnucleus (Sp5O), interpolar subnucleus (Sp5I), and caudal subnucleus (Sp5C). Signal was present on both the ipsilateral and contralateral sides, with no significant differences between two sides. Neutralizing antitoxin significantly reduced cl-SNAP-25 scores in all analyzed subnuclei (Figure 1).

**Figure 2:**
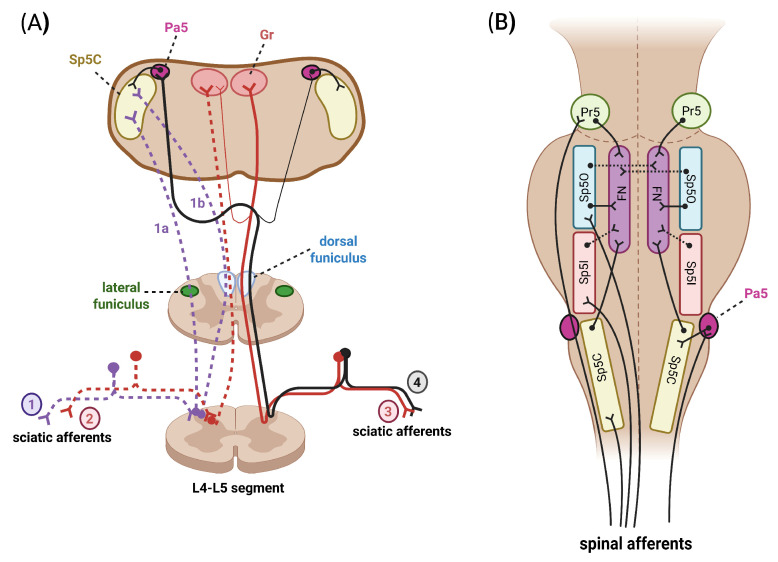
(**A**) Hypothetical ascending neural pathways through which peripherally administered BoNT-A reaches the examined supraspinal regions. After the synapse of the primary afferent neuron (1) with secondary neurons (1a and 1b) forming the spinotrigeminal tract (purple dashed lines), BoNT-A may ascend via ipsilateral projections traveling through the lateral funiculus (1a) to the ventral regions of the caudal trigeminal subnucleus (Sp5C), as well as via projections arising through the dorsal funiculus (1b) to the dorsal regions of Sp5C. A potential direct pathway to the trigeminal complex consists of primary sciatic afferent fibers (4) that ascend through the dorsal funiculus and terminate predominantly in the contralateral (thick black line) paratrigeminal nucleus (Pa5), which in turn sends efferent projections to the Sp5C. The gracile nucleus (Gr) receives spinal afferents forming the gracile tract of the dorsal column. This tract is divided into a direct projection (3), which primarily terminates at the level of the ipsilateral Gr (thick red line), with sparse projections to the contralateral nucleus (thin red line), constituting the direct dorsal column pathway. In contrast, projections that form synapses at the level of the dorsal horn (2) and then ascend ipsilaterally through the dorsal funiculus (red dashed line) constitute the postsynaptic dorsal column pathway. (**B**) Rostrocaudal representation of spinal afferent projections to the nuclei of the trigeminal complex and their interconnections with the facial nucleus (FN). Projections forming the spinotrigeminal tract terminate at the level of the principal sensory nucleus (Pr5) and all three subnuclei (Sp5O, Sp5I, Sp5C) of the spinal trigeminal nucleus. Additional input to the Sp5C is provided by projections originating from the paratrigeminal nucleus (Pa5), which receives direct sciatic afferents. Furthermore, the Pr5 and the subnuclei of the spinal trigeminal nucleus send ipsilateral projections to the FN, with projections from the Sp5I being less frequent (dashed line), while the Sp5O also gives rise to contralateral projections (dashed line) to the FN. Figure is created in BioRender. Farma, A. (2026) https://BioRender.com/4s2vvkq (accessed on 25 February 2026).

As a possible explanation for these observations, we hypothesize that the central occurrence of cl-SNAP-25 signal, as a marker of biologically active BoNT-A, might be attributable to two afferent pathways. The first is the spinotrigeminal tract (Figure 2A), whose projections originate in the spinal cord and terminate at the level of the spinal trigeminal nucleus, ascending through the lateral (Figure 2A-1a) and dorsal (Figure 2A-1b) funiculi. These projections are predominantly present within the Sp5C and enter the Sp5I, Sp5O, and Pr5 via the spinal trigeminal tract [26]. Sporadic direct projections from the dorsal horn ganglia of the lumbar segments L4 and L5 were also found within the Sp5C [27].

Another potential afferent pathway which could explain the occurrence of cl-SNAP-25 immunoreactivity in Sp5C consists of fibers originating from the sciatic nerve that ascend within the dorsal column gracile tract, reaching the contralateral paratrigeminal nucleus (Pa5). This is consistent with predominant contralateral labeling and only transient ipsilateral involvement (Figure 2A-4) observed in tract-tracing studies [28]. Both paratrigeminal nuclei are reciprocally connected and project to the spinal trigeminal nucleus [29,30,31].

Another possible explanation for the occurrence of the cl-SNAP-25 in bilateral trigeminal nuclei is systemic BoNT-A spread from the hind-paw pad. This could account for: (a) similar signal intensity in ipsilateral and contralateral nuclei; and (b) BoNT-A signal occurring predominantly in nuclei with extensive peripheral connections. The bilateral trigeminal nerve may be the major route for traffic to Pr5, Sp5O, Sp5I and Sp5C [32], but other cranial nerves such as the vagal or glossopharyngeal nerve may also contribute to the signal within the trigeminal complex, while the axonal transport via the bilateral facial nerve may be responsible for the signal in the facial nucleus.

Notably, no cl-SNAP-25 was detected in the motor cortex, the source of major corticospinal input to ventral horn [33], or ventral posterolateral nucleus, where secondary sensory neurons project via the spinothalamic tract [34].

Although our previous experiment [15] showed no effect of BoNT-A (7 U/kg, i.pl.) on rota-rod motor performance or visible systemic toxicity (e.g., weight loss), systemic spread from the hind paw after i.pl. administration cannot be completely ruled out, since potential systemic effects of the toxin may be subtle and not measurable by common motor performance tests. However, a slightly higher BoNT-A dose (10 U/kg, onabotulinumtoxinA) injected into the rat knee impaired rota-rod performance, and mildly reduced weight gain rate [35]. Obviously, the presence of cl-SNAP-25 in distant regions does not necessarily imply measurable functional changes at the behavioral level.

### 2.2. Cleaved SNAP-25 Expression Is Brain Region-Selective and Is Attenuated by Antitoxin

Immunohistochemical analysis revealed a region-selective distribution of cl-SNAP-25 following unilateral peripheral BoNT-A application.

#### 2.2.1. Gracile and Facial Nuclei

**Figure 3:**
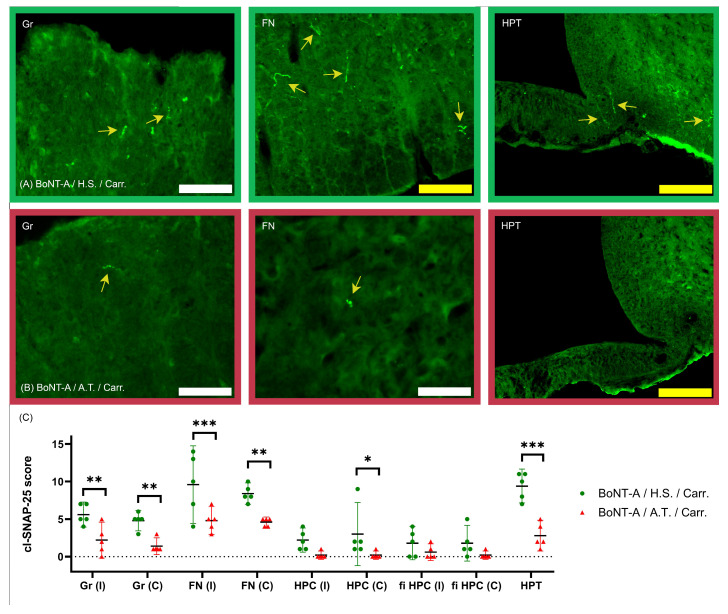
Peripheral BoNT-A induces region-selective supraspinal cl-SNAP-25 immunoreactivity that is reduced by the antitoxin treatment. (**A**) Representative images of ipsilateral BoNT-A proteolytic activity (cl-SNAP-25; green immunostaining indicated by arrows) in the gracile nucleus (Gr), facial nucleus (FN), and hypothalamus (HPT) after unilateral peripheral BoNT-A administration. (**B**) Antitoxin treatment reduced cl-SNAP-25 immunoreactivity across all three regions. Images outlined in green correspond to animals treated with BoNT-A alone, whereas those in red correspond to animals receiving BoNT-A with antitoxin. White scale bars indicate 100 µm, while yellow 200 µm. (**C**) Semi-quantitative analysis of cl-SNAP-25 immunoreactivity in Gr, FN, and HPT. For each animal (N = 5), values were obtained by summing up mean scores from three independent observers. Results are expressed as the mean with 95% CI. Data were analyzed using linear mixed-effects models. *p*-values were adjusted for multiple comparisons using the two-stage linear step-up procedure of Benjamini, Krieger, and Yekutieli to control the false discovery rate (FDR < 0.05). Adjusted *p* < 0.05 was considered statistically significant. * = *p* < 0.05; ** = *p* < 0.01; *** = *p* < 0.001. Figures were processed for brightness and contrast adjustments using Adobe Photoshop 2021 (version 22.0.0; Adobe Systems Incorporated, San Jose, CA, USA). Abbreviations: Gr = gracile nucleus; FN = facial nucleus; HPC = hippocampus; fi HPC = fimbria of hippocampus; HPT = hypothalamus; I = ipsilateral; C = contralateral; H.S. = horse serum; A.T. = antitoxin to BoNT-A; Carr. = carrageenan.

Cleaved SNAP-25 immunoreactivity was detected in the facial and the gracile nuclei. In both the brainstem regions, signaling was present on the ipsilateral and contralateral sides, where neutralizing antitoxin significantly reduced cl-SNAP-25 bilaterally (Figure 3).

The presence of the signal within both Gr could be the result of BoNT-A transport primarily via the postsynaptic (Figure 2A-2) and direct tracts (Figure 2A-3) of the dorsal column. Specifically, fibers originating from regions below the gelatinous substance of the spinal cord at the L4–L6 segment levels terminate within the Gr, forming an integral part of the postsynaptic tract of the dorsal column [36]. Lumbar projections of this tract are distributed throughout the entire Gr [37]. On the other hand, the direct tract consists of direct projections that do not synapse at the level of the dorsal horn of the spinal cord [38]. These afferent fibers of the sciatic nerve ascend within the gracile fasciculus and terminate at the level of the ipsilateral, and to a lesser extent the contralateral Gr [39]. This could possibly explain the detection of the signal within the contralateral Gr in the present study. Most of these fibers, whose neuronal cell bodies are located within the dorsal horn ganglia of the L4 and L5 segments, are responsible for the cutaneous innervation of the rat paw [27], and were found to be immunopositive for substance P, calcitonin gene-related peptide (CGRP), galanin, and nitric oxide (NO) [40].

The spinal trigeminal nucleus and gracile nucleus are key brainstem processing centers for somatosensory information from the orofacial region and hind paw/caudal body, respectively [41,42]. Detection of cl-SNAP-25 in both nuclei after unilateral peripheral BoNT-A administration suggests that the toxin may modulate sensory processing at multiple supraspinal levels. However, whether this contributes to its segmental antinociceptive effect, as observed in our previous behavioral experiment (unpublished results), deserves further investigation.

Projections originating from the lumbar segments L4–L6 around the central canal of the spinal cord ascend to the pons, including the facial nucleus, with these projections primarily crossing to the contralateral side [43]. These projections could explain the detection of the signal within both the ipsilateral and contralateral FN. However, due to the interconnection of the FN with the trigeminal nuclei (Figure 2B) [44], we speculate that the entry of BoNT-A into FN could occur through these regions. Specifically, Sp5C projects to the ipsilateral part of the FN, while projections from the Sp5I are rarer and distributed across the ipsilateral lateral, dorsal, and medial portions. Projections from the Sp5O, unlike the other two subnuclei, are bilateral and terminate in the medial part of the FN [45]. Thus, spinal trigeminal neurons project ipsilaterally to the lateral FN and bilaterally to its medial part, with this trigeminofacial connection forming part of the corticofacial motor loop for orofacial sensory–motor integration [46].

#### 2.2.2. Hypothalamus and Hippocampus

Analysis across the entire hypothalamic (HPT) region revealed cl-SNAP-25 mainly in the median eminence (ME), with sporadic signals in the arcuate nucleus (ARC) and ventromedial hypothalamic nucleus (VMH). Sections from antitoxin-treated animals showed significant signal attenuation (Figure 3). Anatomically, BoNT-A could enter the HPT via the spinohypothalamic tract, which conveys somatosensory and visceral information from spinal cord deep dorsal horn layers, the lateral spinal nucleus, the superficial dorsal horn, the central canal area, and Sp5C to medial and lateral HPT regions [38,47,48]. Furthermore, due to a more permeable blood–brain barrier at these sites, toxin distribution via circulation cannot be ruled out.

Direct spinal afferents to the ARC, VMH, or ME have not been reliably demonstrated in major tract-tracing studies [46,49], so the presence of the cl-SNAP-25 in these regions is unlikely to reflect direct synaptic spinal input. In theory, these findings may be explained by indirect central pathways within hypothalamic circuits, as ARC and ME are tightly interconnected with other hypothalamic nuclei that do receive spinal or brainstem inputs, such as the paraventricular nucleus (PVN), dorsomedial hypothalamic nucleus (DMH), and lateral hypothalamic area [50,51,52].

It is important to emphasize that both the ARC and ME are characterized by a porous blood–brain barrier, allowing circulating molecules, such as hormones, peptides or cytokines, greater access to neural tissue [53]. This raises the possibility that peripheral BoNT-A could reach these regions via circulation rather than through axonal transport, although this was not directly investigated in the present study and will need to be addressed in future experiments. In contrast, the VMH is well protected by an intact blood–brain barrier but does not receive direct spinal projections [54], suggesting that the cl-SNAP-25 detected in this nucleus may reflect indirect intrahypothalamic connectivity.

Within the hippocampus (HPC), the cl-SNAP-25 immunoreactivity was detected bilaterally in the fimbria as well as in the other hippocampal subregions. The antitoxin reduced the expression of cl-SNAP-25 on both sides, but this effect was significant on the contralateral side of HPC only (Figure 3).

A potential route for BoNT-A transport to HPC could involve projections from the trigeminal complex and HPT. The literature suggests minor trigeminal complex projections to the rat HPC [55]. HPT-HPC connections form a loop mediating behavioral responses, with the supramammillary nucleus primarily projecting to posterior dorsal HPC (less to ventral HPC) and the submamillothalamic nucleus projecting exclusively to dorsal HPC (with minor contralateral input) [56,57]. Given that the HPC fimbria is a major route for afferent and efferent fibers of the hippocampal formation [58], the cl-SNAP-25 signal presence there is unsurprising.

In addition, HPC has a relatively more permeable blood–brain barrier than other brain regions, with permeability increasing with age in adult rats [59,60]. In the present study, rats were 4–5 months old [15]. Thus, as in the hypothalamus, systemic toxin spread and blood–brain barrier traversal cannot be ruled out as explanations for the observed findings. Further experiments should address this possibility.

### 2.3. Other Brain Regions with Sparse cl-SNAP-25 Occurrence

In contrast to the above-discussed cleaved SNAP-25 occurrence in the regions with a high density of intermediate to intensely fluorescent fibers, the following regions showed only low-intensity signals. These were limited to a few sections and lacked dense or widespread distribution. Therefore, these findings should be interpreted cautiously, bearing in mind the methodological limitations of the present study (see Section 2.6). Therefore, these incidental observations were not assessed using the proposed semi-quantitative scale and were excluded from statistical analyses. However, some of them are worth considering in the context of BoNT-A’s central antinociceptive effect.

Cleaved SNAP-25 immunoreactivity was sparsely detected in subregions of the solitary tract (Sol), cuneate nucleus (Cu), external cuneate nucleus (ECu), nucleus X (X), and the medullary matrix region (Mx). Within the pons and midbrain, weak labeling was occasionally observed in the locus coeruleus (LC), superficial gray layer (SuG) and periaqueductal gray (PAG).

Although sparse, cl-SNAP-25 detection in PAG suggests BoNT-A access via two potential routes: (1) direct spinomesencephalic tract fibers from the spinal dorsal horn [61], or (2) spinohypothalamic tract collaterals primarily targeting the hypothalamus (HPT) [62]. The locus coeruleus (LC) receives PAG afferents and sparse direct spinal inputs from the dorsal horn, intermediate gray matter, and lamina X [63,64].

Trigeminal inputs to the superior colliculus arise primarily from Pr5 and Sp5I, terminating predominantly in intermediate/deep layers (IV–VI) of the colliculus with contralateral preference, rather than forming major direct inputs to the SuG. Similarly, ascending spinal nociceptive projections via spinotectal/spinomesencephalic tracts target mainly caudal colliculus intermediate layers, with limited SuG termination [65,66,67,68]. These patterns might indicate that BoNT-A reaches SuG via ascending trigeminal/spinal pathways, though superficial layer involvement appears limited.

Altogether, based on these observations it can be tentatively suggested that the involvement of dorsal medullary nuclei, pons and midbrain in the BoNT-A central effects remains uncertain and requires further, more detailed investigation in future studies.

### 2.4. Brain Regions with Not Detected cl-SNAP-25 Signal

No cl-SNAP-25 immunoreactivity was detected in the parabrachial pigmented nucleus, thalamus, amygdala and sensory cortex.

In the control group receiving physiological saline instead of BoNT-A, no cl-SNAP-25 immunoreactivity was detected in any examined brain region, confirming that the signals observed in the BoNT-A-treated groups reflect specific toxin activity rather than artifacts. The specificity of the antibody was validated previously by the manufacturer [69,70] and confirmed by our previous experiments that employed the same immunohistochemical protocol [14,15,71].

### 2.5. Interpretation of Supraspinal cl-SNAP-25 Detection and Open Questions

This paper represents the first investigation of the distribution of BoNT-A, administered into the rat hind-paw pad, within brain regions beyond the first synapse at the spinal cord. Bilateral detection of cl-SNAP-25 in the analyzed supraspinal regions could indicate retroaxonal transport of the toxin from the periphery, as well as inter-synaptic transfer and neuronal distribution within distant regions. These results are consistent with previous in vitro studies in which cl-SNAP-25 was detected in neurons located two or more synapses away from the toxin’s application site [71,72]. Notably, the cl-SNAP-25 signal was attenuated when a specific BoNT-A-neutralizing antitoxin was applied, suggesting that central toxin distribution depends on trans-synaptic neuronal transport, although the exact sites of trans-synaptic trafficking remain unknown. However, possible alternative routes, including haematogenous toxin dissemination followed by its entry into the CNS at sites of increased blood–brain barrier permeability, could also contribute.

In our previous experiments [15], cl-SNAP-25 signals were detected in the dorsal horns of the L4–L5 spinal segments, corresponding to the entry point of sciatic projections into the spinal cord. The mechanism of BoNT-A-mediated analgesia has thus far been primarily attributed to interference with pain signal processing at the first synapse in the dorsal horn [9]. Nevertheless, the contribution of supraspinal distribution to central antinociceptive effects cannot be entirely excluded. Based on the present results, in which only sparse, if any, cl-SNAP-25 signal was detected in nociceptive brain regions such as the LC and PAG; and with a comparatively greater signal observed in the hypothalamus, supraspinal sites likely play a negligible role in the toxin’s antinociceptive action.

However, stronger cl-SNAP-25 signals observed in the trigeminal complex, facial nucleus, and gracile nucleus suggest that BoNT-A may affect other sensory and information-processing pathways, warranting further in-depth analysis. As hypothesized, BoNT-A could reach these nuclei via ascending neural pathways, either through direct retrograde axonal transport along primary afferents from the hind paw or/and via transcytosis into secondary ascending neurons at the spinal level, allowing further supraspinal distribution (it is presumed that the toxin exits the primary afferent fibers and subsequently enters second-order neurons). Furthermore, contralateral cl-SNAP-25 signals may result from cross-midline projections of ascending afferents at spinal entry or from collateral branches of neurons. In hypothalamic regions, cl-SNAP-25 presence most likely reflects indirect central pathways and region-specific blood–brain barrier accessibility rather than direct spinal afferent input.

A possible contribution of systemic spread of the toxin, followed by retrograde axonal transport within distant peripheral nerves, may also explain the occurrence of cl-SNAP-25 in the brain. This mechanism could account for the bilateral distribution of cl-SNAP-25 signals, as well as the fact that many brain regions receive direct afferent or efferent input via peripheral nerves, such as the trigeminal or facial nerves. In contrast, some regions located two synapses away that receive substantial spinal input (e.g., the ventral posterolateral thalamic nucleus and sensory cortex) do not show detectable cl-SNAP-25 signals.

The presence of cl-SNAP-25 in the median eminence (ME) of the hypothalamus strongly suggests systemic hematogenous toxin spread as the explanation for this specific localization. Interestingly, potential effects of the toxin on nerve terminals in the ME, which regulate the release of neuropeptides such as corticotrophin-releasing factor, could contribute to BoNT-A’s antidepressant action [73].

Furthermore, the partial blockade of cl-SNAP-25 by antitoxin in the CNS may reflect that some of the toxin molecules already reached the CNS within 24 h from toxin injection, as opposed to the longer trans-synaptic traffic over two or more synapses, which would otherwise allow the antitoxin a greater opportunity to fully inhibit central cl-SNAP-25 accumulation.

### 2.6. Study Limitations

Several limitations should be considered when interpreting the present findings. First, cl-SNAP-25 detection in certain supraspinal regions was sporadic, in some cases limited to a single fiber in two–three animals (i.e., one fiber across six–nine analyzed sections). These observations were therefore evaluated descriptively and should be interpreted with great caution. Second, although the semi-quantitative scoring approach is suitable for assessing regional distribution patterns of BoNT-A proteolytic activity, which was the main scope of the present study, it does not provide direct quantification of toxin concentration or enzymatic activity. Thus, the semi-quantitative scoring approach represents a limitation of the study and may restrict the level of precision compared to more advanced quantitative image analysis methods.

Moreover, while antitoxin treatment reduced cl-SNAP-25 signals in multiple regions, this approach cannot fully distinguish between axonal, trans-synaptic, or hematogenous routes of toxin transport, particularly in hypothalamic areas characterized by reduced blood–brain barrier integrity. As these important aspects fall beyond the scope of the present study, future research should address these questions using experimental approaches specifically designed to differentiate BoNT-A neuronal transport from potential systemic dissemination.

Another possible explanation for cl-SNAP-25 detection in supraspinal regions is the transport of cleaved SNAP-25 fragments generated peripherally by BoNT-A, which may travel along neuronal pathways to distant sites. Thus, cl-SNAP-25 immunoreactivity may reflect proteolytic activity occurring both at peripheral and central levels. However, reduction in central cl-SNAP-25 signal by BoNT-A-specific intrathecal antitoxin cannot be explained by this possibility. Nevertheless, distinguishing between transported toxin and transported cleavage product remains an important challenge, and further studies are necessary to fully resolve their respective contributions.

Finally, this study examined a single dose and time point following peripheral BoNT-A administration. Future investigations assessing different doses, temporal dynamics, and peripheral antitoxin application will be necessary to fully elucidate the mechanisms, distribution patterns, and functional relevance of supraspinal BoNT-A occurrence.

## 3. Conclusions

This study provides the first evidence that a single unilateral peripheral administration of BoNT-A into the rat hind paw leads to cl-SNAP-25 detection in multiple supraspinal regions. The bilateral distribution of the signal, together with its attenuation following BoNT-A-neutralizing antitoxin treatment, suggests that BoNT-A gains access to distant brainstem, midbrain, and hypothalamic structures, possibly via retrograde and trans-synaptic transport along ascending sensory pathways from the periphery. However, with the present experimental approach, the contribution of systemic circulatory distribution cannot be excluded. Therefore, both neuronal transport and systemic dissemination remain plausible and non-mutually exclusive explanations for the observed findings. Overall, these findings highlight the need for further investigations to clarify the pathways of BoNT-A distribution from the peripheral application site, determine the functional significance of its central presence, and assess the implications for therapeutic efficacy and potential adverse effects.

## 4. Materials and Methods

### 4.1. Animals and Experimental Design

All experimental procedures were approved by the Ethical Committee of the University of Zagreb School of Medicine and the Croatian Ministry of Agriculture (permit no. 386/2023) and were conducted in compliance with EU Directive 2010/63/EU and ARRIVE 2.0 guidelines [74].

The present work represents an analysis of the brain tissues harvested from the animals (adult male Wistar rats, aged 4–5 months and weighing 450–550 g, housed at the Department of Pharmacology, University of Zagreb School of Medicine, Croatia) included in our recently published experiments [15]. According to the 3R principle (replacement, reduction, and refinement), no additional animals were used for the purpose of this study; instead, in the present experiment, the brain tissues were collected along with the spinal cord previously subjected to immunohistochemical analyses, to investigate the supraspinal distribution of the cleaved SNAP-25 after toxin’s unilateral application into the rat hind-paw pad (7 U/kg). Therefore, the experimental protocol, animal grouping, drug administration procedures, carrageenan-induced inflammatory pain model and immunohistochemical procedures for the detection of cl-SNAP-25, including the tissue collecting and antibody staining, have been described in detail in our previous study [15].

Briefly, here we compare brain tissue from three groups of rats in a carrageenan-induced inflammatory pain model. Control animals received intraplantar (i.pl.) saline, followed the next day by an intrathecal (i.t.) injection of horse serum (HS). Experimental Group I received i.pl. BoNT-A [onabotulinumtoxinA-Botox^®^ (Allergan Inc., Irvine, CA, USA), 7 U/kg, 20 µL] and 24 h later, an i.t. HS (Gibco, ThermoFisher Scientific, Waltham, MA, USA) injection. Experimental Group II received the same i.pl. BoNT-A dose but were given an i.t. 2 IU/10 µL BoNT-A antitoxin (lyophilized polyclonal equine IgG-based BoNT-A antitoxin—from National Institute for Biological Standards and Control, Potters Bar, UK, NIBSC code 14/174, provided by Thea Sesardic, PhD, and Paul Stickings, PhD) instead of HS. Six days after the initial injections, 2% carrageenan (100 µL; λ-Carrageenan^®^, Sigma-Aldrich, St. Louis, MO, USA) was administered intraplantarly into both hind paws to provoke mechanical hyperalgesia, which peaked 3–5 h post-injection. After behavioral measurements, the animals were deeply anesthetized by i.pl. 70/7 mg/kg of ketamine/xylazine (Ketamidor^®^ 10%, Richter Pharma AG, Wels, Austria/Xylased Bio^®^ 20 mg/mL, Bioveta, Ivanovice na Hané, Czech Republic) and then euthanized by transcardial perfusion with saline (250 mL), followed by fixative consisting of 4% paraformaldehyde in 0.01 M phosphate-buffered saline (PBS; pH = 7.4, 250 mL) [21]. After dissection, the brain tissue was cryoprotected overnight in a 15% sucrose-fixative solution and then transferred to 30% sucrose in PBS. Once the tissue had sunk, it was removed, briefly blotted on filter paper to eliminate excess liquid, and stored at −80 °C.

### 4.2. Immunohistochemistry

Coronal sections (35 µm) of the examined brain regions were cut using cryostat (Leica CM 1950, Wetzlar, Germany) and collected into PBS-filled wells. Prior to sectioning, the left side of the tissue was carefully punctured with a tuberculin syringe needle to mark the contralateral side to the BoNT-A injection. Sections of the examined brain regions of 5 rats per animal group were taken for free-floating immunohistochemical analysis. Immunohistochemical staining was conducted as stated in the manufacturer’s guidelines for goat anti-rabbit Alexa Fluor™ 488 Tyramide SuperBoost™ Kit (Invitrogen by Thermo Fisher Scientific, Eugene, OR, USA). Non-affinity purified rabbit antiserum anti-SNAP-25 (1–197) (National Institute for Biological Standards and Control, Potters Bar, UK; kindly provided by Thea Sesardic, PhD) validated in previous studies [69,70], diluted 1:8000 in 1% NGS (normal goat serum), was incubated overnight in wells with tissue sections at room temperature. Then, sections were rinsed and placed on glass slides with anti-fading agent (Fluoroshield with DAPI, Sigma-Aldrich, St. Louis, MO, USA) and left overnight at +4 °C.

### 4.3. Visualization of cl-SNAP-25 Protein Distribution

Brain tissue samples from five animals per treatment group were examined immunohistochemically for the cl-SNAP-25 signal in the following regions: the trigeminal complex subnuclei (Pr5—principal sensory nucleus of the trigeminal nerve; Sp5O—oral, Sp5I—interpolar and Sp5C—caudal subnucleus), facial nucleus (FN), gracile nucleus (Gr), nucleus X (X), matrix region of the medulla (Mx), different regions of the nucleus of the solitary tract (Sol), external cuneate nucleus (ECu), locus coeruleus (LC), periaqueductal gray (PAG), parabrachial pigmented nucleus of the ventral tegmental area (PBP), superficial gray layer of the superior colliculus (SuG), hypothalamus (HPT), hippocampus (HPC), thalamus (Th), amygdala (Amyg) and primary somatosensory cortex (S1).

From each animal, three randomly selected transverse brain tissue sections were analyzed using a fluorescence microscope (Axio Observer 7, Zeiss, Oberkochen, Germany) equipped with a digital camera (Axiocam 305 with a 0.63× adapter).

Signal intensity was assessed using a semi-quantitative immunohistochemical scoring method focused on the density and fluorescence intensity of cl-SNAP-25-positive nerve fibers. Fluorescence signals within the examined regions were independently evaluated by three observers who were blinded to the treatment conditions. Scoring was performed on a scale from 0 to 5, representing increasing fiber density and signal intensity (Table S1). Score 0 indicated no evident signal, scores 1–2 indicated sparse labeling with only a few individual cl-SNAP-25 positive fibers, whereas scores of 3–5 reflected regions with a high density of fibers showing moderate to very strong fluorescence intensity (Table S1 and Figure S2 in Supplementary Materials). Three sections of each animal were scored individually, by each of three observers, and the scores were summed to obtain an animal-specific cumulative score. Three sections from each animal were scored individually by each of the three blind observers. For each observer, the mean score across the three sections was calculated, and the resulting mean values from all observers were then summed to obtain an animal-specific cumulative score.

In regions showing low signal intensity, limited to a few sections and lacking dense or widespread distribution, signal assessment using the proposed semi-quantitative scale was not performed.

### 4.4. Data Analysis

Statistical analysis and graph drawing were performed using GraphPad Prism 8 (version 8.01, GraphPad Software, Inc., La Jolla, CA, USA). Data were analyzed using the linear mixed-effects models. Two separate analyses were conducted: one to evaluate differences between the ipsilateral and contralateral sides within the same brain region, and a second to assess treatment-dependent effects within the ipsilateral and contralateral sides separately. In all models, treatment group and/or injection side were included as fixed factors, and animals (rat identifier) were included as a random intercept to account for repeated measurements within subjects. *p*-values were adjusted for multiple comparisons using the two-stage linear step-up procedure of Benjamini, Krieger, and Yekutieli to control the false discovery rate (FDR < 0.05). Adjusted *p*-values < 0.05 were considered statistically significant.

## Data Availability

The original contributions presented in this study are included in this article and the Supplementary Material. Further inquiries can be directed to the corresponding author.

## Supplementary Materials

The following supporting information can be downloaded at https://www.mdpi.com/article/10.3390/toxins18060261/s1: Immunohistochemical pictures of control sections (Figure S1). Semi-quantitative scoring of cleaved SNAP-25 immunoreactivity (Table S1). Six representative immunohistochemical images (Figure S2).

## Abbreviations

The following abbreviations are used in this manuscript:

ARC	Arcuate nucleus
A.T.	Antitoxin to BoNT-A
BoNT-A	Botulinum toxin type A
C	Contralateral
cl-SNAP-25	Cleaved SNAP-25
CNS	Central nervous system
Cu	Cuneate nucleus
Ecu	External cuneate nucleus
FN	Facial nucleus
Gr	Gracile nucleus
HPC	Hippocampus
HPT	Hypothalamus
HS	Horse serum
I	Ipsilateral
i.pl.	Intraplantar
i.t.	Intrathecal
LC	Locus coeruleus
ME	Median eminence
Mx	Medullary matrix region
Pa5	Paratrigeminal nucleus
PAG	Periaqueductal gray
Pr5	Principal sensory nucleus
SNAP-25	Synaptosomal-associated protein of 25 kDa
Sol	Solitary tract
Sp5C	Caudal trigeminal subnucleus
Sp5I	Interpolar trigeminal subnucleus
Sp5O	Oral trigeminal subnucleus
SuG	Superficial gray layer
Th	Thalamus
VMH	Ventromedial hypothalamic nucleus
X	Nucleus X
