# Supplementary material for: Regional Brain Localization of Botulinum Toxin Type A-Truncated Synaptosomal-Associated Protein 25 After Injection into the Rat Hind Paw
Source: Toxins (Basel). 2026 Jun 9;18(6):261. doi: 10.3390/toxins18060261 (PMC13308166; doi:10.3390/toxins18060261)
Supplement: Supplementary file 1 [file toxins-18-00261-s001.zip › toxins-4314512-supplementary.pdf]

Supplementary materials: Regional brain localization of botulinum toxin type A-induced synaptosomal-associated protein 25 cleavage after injection into the rat hind paw

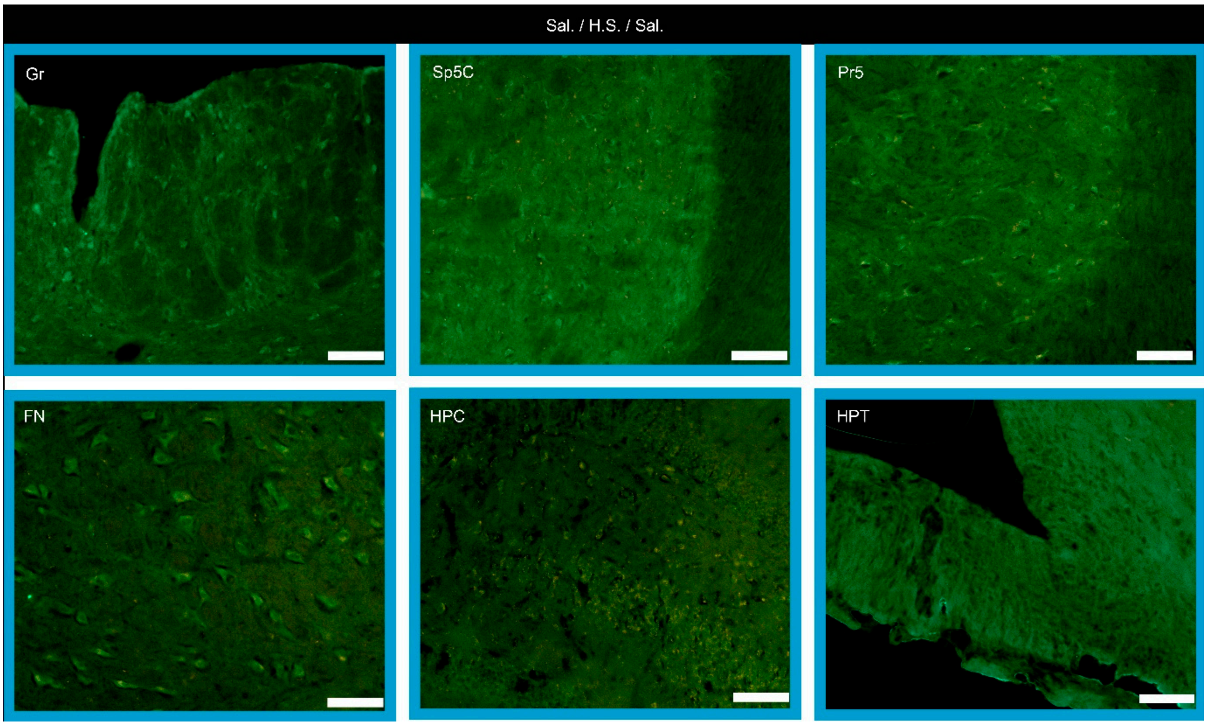

Figure S1. Immunohistochemical pictures of control sections—absence of nonspecific cl-SNAP-25 immunofluorescence signal. *Representative fluorescence micrographs of control animals receiving unilateral intraplantar (i.pl.) saline (day 0) followed by the intrathecal horse serum administration (day 1), and saline i.pl. treatment (day 6). The analyzed regions included the gracile nucleus (Gr), spinal trigeminal nucleus caudalis (Sp5C), principal sensory trigeminal nucleus (Pr5), facial nucleus (FN), hippocampus (HPC), and hypothalamus (HPT). No cl-SNAP-25-positive fibers or specific immunofluorescence signals were detected in any examined region, and no nonspecific staining resembling cl-SNAP-25 immunoreactivity was observed. Scale bar: 100 µm. Abbreviations: Sal. = saline; H.S. = horse serum.*

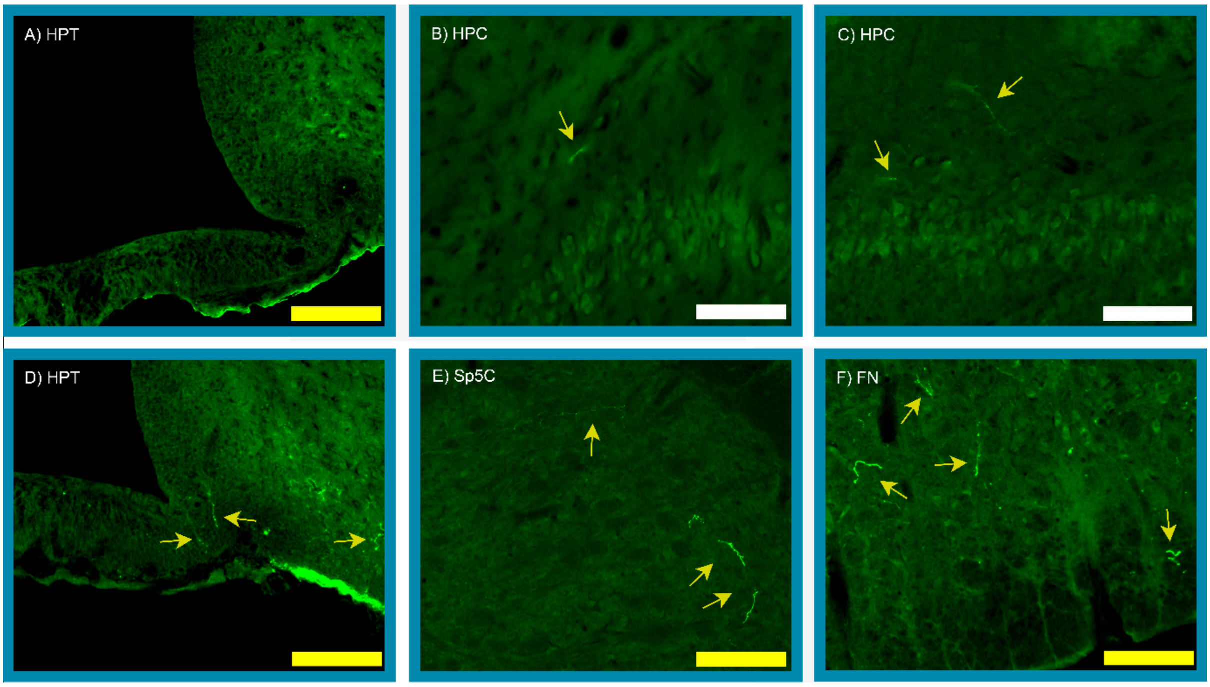

Figure S2. Semi-quantitative scoring of cleaved SNAP-25 immunoreactivity. Six representative immunohistochemical images (A-F) illustrate the scoring system for cleaved SNAP-25-positive fibers. The first image (A) shows the hypothalamus (HPT) of an antitoxin-treated rat with no detectable signal (score 0). The second (B) and third (C) images show hippocampus (HPC) labelling after BoNT-A administration, with sparse ipsilateral (score 1) and a slightly higher contralateral (score 2) signal. The fourth image (D) represents moderate cl-SNAP-25 immunoreactivity (score 3) in the hypothalamus (HPT) of a BoNT-A-treated rat. The fifth image (E) shows strong labelling (score 4) in the ipsilateral spinal trigeminal nucleus pars caudalis (Sp5C), and the sixth image (F) shows very strong labelling (score 5) in the ipsilateral facial nucleus (FN). Yellow scale bars indicate 10× magnification (200 μm), and white scale bars indicate 20× magnification (100 μm).

Table S1. Semi-quantitative scoring of cleaved SNAP-25 immunoreactivity.

Score	Description
0	No detectable cleaved SNAP-25-positive fibers
1	Presence of a single cleaved SNAP-25-positive fiber
2	Presence of two to three individual cleaved SNAP-25-positive fibers
3	Moderate signal with a high density of fibers and intermediate fluorescence intensity
4	Strong signal with a high density of brightly fluorescent fibers
5	Very strong signal with a very high density of intensely fluorescent fibers
